# Creating accessible evidence bases: Opportunities through the integration of interactive tools into literature review synthesis

**DOI:** 10.1016/j.mex.2021.101558

**Published:** 2021-10-25

**Authors:** Sebastian Scheuer, Jessica Jache, Luca Sumfleth, Thilo Wellmann, Dagmar Haase

**Affiliations:** a Humboldt-Universität zu Berlin, Geography Department, Landscape Ecology Lab, Unter den Linden 6, Berlin 10099, Germany; b Department of Computational Landscape Ecology, Helmholtz Centre for Environmental Research -UFZ, Leipzig 04318, Germany

**Keywords:** Dissemination, Systematic literature review synthesis, Interactive visualization and mapping, Data science, Structured data, Web application, Dashboard

## Abstract

The COVID-19 pandemic has shown that an immediate access to relevant information is key for timely interventions and forming of public opinion and discourse. In this regard, dashboards present themselves as invaluable tools for the democratization of data and for the creation of accessible evidence bases. Building on this momentum, it is proposed to integrate interactive means such as dashboards into academic literature review synthesis, in order to support the summarization, narration, and dissemination of findings, and furthermore, to increase transparency and support the transferability and comparability of findings. Exemplified for a systematic literature review on urban forests as nature-based solutions,

• Key functionalities, requirements and design considerations for the development of dashboards for use in academic literature reviews synthesis are identified.
• An application architecture that embeds dashboard development into an R workflow is presented, with emphasis on the steps needed to transform the data collected during the review process into a structured form.
• Technical and methodological means for the actual dashboard implementation are highlighted, considering the identified key functionalities and requirements.

## Graphical abstract

**Figure:**
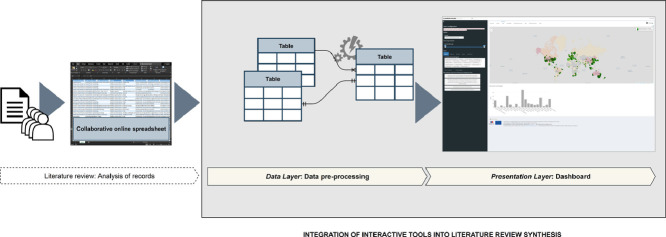

Specifications table

**Table:** 

Subject Area	Environmental Science
More specific subject area	*Knowledge and data dissemination, data science*
Method name	*Integration of interactive dashboards into academic literature review synthesis*
Name and reference of original method	*The proposed method does not build on a specific method per-se, but seeks to expand on or integrate methodologies for conducting literature reviews, for data structuring and data normalization, and interactive data visualization.* *Methods for selected review types and subsequent requirements: M. J. Grant, A. Booth, A typology of reviews: an analysis of 14 review types and associated methodologies, Health Information and Libraries Journal. 26 (2009) 91–108.* *Methods regarding data structuring and data normalization: T. Teorey, S. Lightstone, T. Nadeau, Normalization, Database Modeling and Design, 4th ed., Morgan Kaufmann, Burlington, MA, USA, 2006.* *Interactive data visualization: R. Matheus, M. Janssen, D. Maheshwari, Data science empowering the public: Data-driven dashboards for transparent and accountable decision-making in smart cities, Government Information Quarterly. 37 (2020) 101284.*
Resource availability	*The dashboard can be publicly accessed at*https://review.clearinghouseproject.eu*.*

## Background

To make cities more livable, to support climate change adaptation through increasing resilience, and to address related socio-environmental challenges such as the improvement of human health and well-being, urban areas ought to become more sustainable, greener, and thereby, more environmentally just. To meet these demands, evidence-based, effective interventions within the urban space, e.g., greening, are key. Devising such interventions relies on appropriate indicators, e.g., for an adequate assessment of their impacts, that should meet the criteria of: (i) credibility and validity, i.e., the scientific adequacy of arguments and data with respect to the indicandum; (ii) salience, i.e., the relevancy of corresponding assessments, including aspects of scalability and transferability; and (iii) legitimacy, i.e., the application of a rigorous scientific process to generate information and knowledge [1].

Credibility, salience, and legitimacy are backed through expert judgement and empirical evidence [1]. Systematic literature reviews (SLR) are a common means for compiling evidence bases from the body of scientific literature. Various guidelines, checklists, and frameworks support SLR, e.g., Enhancing the Quality and Transparency Of health Research (EQUATOR), Enhancing Transparency in Reporting the synthesis of Qualitative research (ENTREQ), or Preferred Reporting Items for Systematic reviews and Meta-analyzes (PRISMA) etc. [2]. PRISMA, for example, recommends items for reporting in SLR that include rationale and objectives, eligibility criteria, sources of literature and search strategy, variables and other data items, as well as methods for reporting of findings [3]. For a successful dissemination of findings, systematic, qualitative and/or quantitative summarizations and presentations of results are also crucial aspects of SLR, often conducted in the form of scientific publications [4]. In the context of nature-based solutions (NBS), i.e., (green-blue) infrastructures for the simultaneous delivery of environmental, social, and economic benefits and for increasing resilience towards natural hazards and climate change [5], this becomes particularly relevant for the promotion, transferability and comparatively of SLR outcomes, to support adoption and mainstreaming of NBS for tackling the involved socio-economic-ecological challenges. In this regard, Dushkova & Haase have previously identified the need for novel, synergistic data-gathering techniques, and for the establishment of innovative, structured data and knowledge platforms [6]. Such data and knowledge platforms may also help in lifting existing information barriers, thus facilitating empirical evidence to gain attention from decision-makers, stakeholders, and the public, through democratizing the access to findings and data [7,8].

With respect to the development of corresponding platforms, dashboards present credible opportunities for their realization, as the disciplines of their origin, i.e., data science and business intelligence, are as well concerned with the presentation, interpretation and sharing of data [9]. To facilitate these tasks, dashboards should provide their users with a clear presentation, means of customization, e.g., of the visualizations used, support of different views on data and information drilling—i.e., operations and transformations on data and presentation of the data on different levels of detail—inclusive of update and/or transfer of data. Subsequently, dashboards are understood as specifically interactive visualizations of consolidated data both for a specific context and/or purpose [9], [10], [11]. Through their interactivity and design, dashboards provide various strategic and operational benefits in line with previously identified needs [7], [8], [9] including orientation towards different user groups, enabling participation and multidisciplinary investigation, transparency, trustworthiness, accountability, effective dissemination of information, and an immediate access to data [9].

These benefits of dashboards crystallized prominently during the COVID-19 pandemic [12]. From the early onset of the pandemic, various dashboards emerged for the dissemination of data at global [13], regional or (sub-)national level [14]. These dashboards enabled the whole world to access data on and monitor viral activity through multiple parameters in near real-time, and in doing so, fulfilled the promise of democratization the access to data and enabled a re-purposing of data by academia and the public to establish additional perspectives on the pandemic situation, e.g., by the integration of socio-economic contexts [12]. Acting on this momentum, the specific integration of dashboards into literature reviews such as SLR or rapid reviews [15] is proposed as a means of reporting and narration, i.e., for a systematic, interactive, immediate, and user-oriented presentation and dissemination of findings. Thereby, as proven in the context of the COVID-19 pandemics, the accessibility to evidence bases shall be increased to contribute to credibility, salience, and legitimacy of research.

In the following, the implementation of such a proposed dashboard is described for an exemplary case for the CLEARING HOUSE project [16] on urban forests as nature-based solutions [17], with a focus on technical-methodological aspects and the dashboard realization.

## Method details

**Fig. 1:**
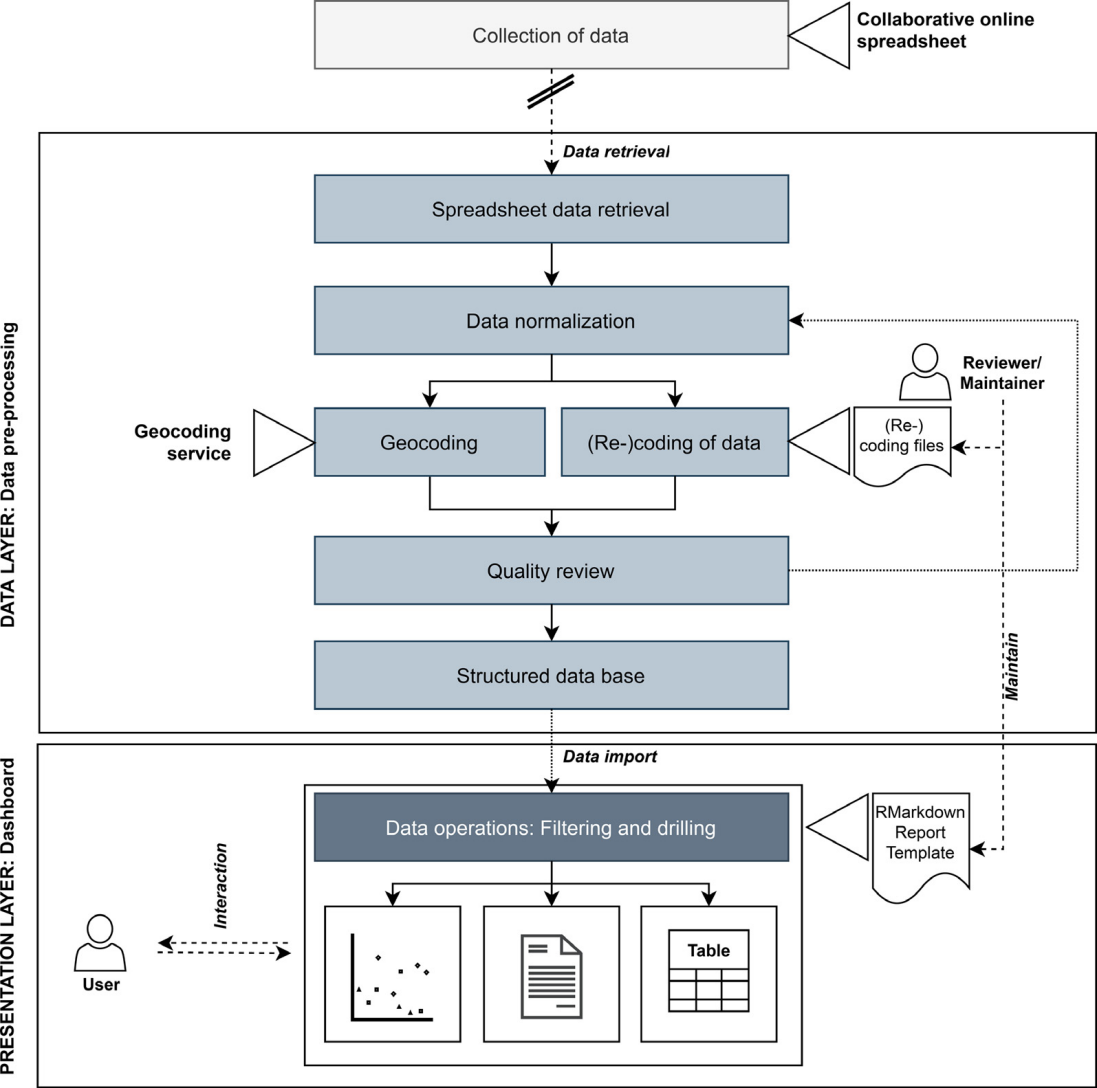
Proposed dashboard implementation as a loosely coupled two-tier architecture comprising a data layer and a presentation layer. The data layer represents the workflow established for the transformation of the unstructured/semi-structured spreadsheet data into the structured data base for use in the dashboard. The presentation layer imports this data base and implements the functionality required for user interaction, presentation, etc.

As shown in Fig. 1, the proposed approach does conceptually represent a two-tier architecture including a data layer that implements the necessary data pre-processing, and a loosely coupled presentation layer that comprises the actual dashboard implementation. The architecture is entirely implemented and embedded into the R statistical language [18].

### Data layer

In line with the needs previously identified by Dushkova & Haase [6], the data pre-processing is conducted to transform unstructured and/or semi-structured data, as collected through the review process, e.g., in the form of a collaborative online spreadsheet, into a structured data base. In so doing, data is prepared for use in the presentation layer (Fig. 1).

The process of data pre-processing includes, first, the normalization of retrieved spreadsheet data (Fig. 1). This is done by the application of normal forms commonly used for the structuring of relational databases [19]. Here, to address the most common data anomalies, the first three normal forms—1NF, 2NF, and 3NF, respectively—are considered. Hence, to achieve 1NF, data is first transformed into atomic values, i.e., value items being broken down to the smallest feasible level of information, to avoid redundancies. Then, primary keys are created per analyzed record so that all non-key data items are fully dependent on this primary key, thereby achieving 2NF. 3NF is subsequently achieved through re-structuring of the data to avoid transitive data dependencies [19]. Essentially, data normalization results in a structured set of data tables with atomic, non-transitively but fully primary-key dependent values, e.g., table(s) of contextual challenge, case study country, case study city, measurement sites etc. Thereby, cardinality relationships present in the data are also addressed, e.g., one-to-one, one-to-many, or many-to-many relationships [19], which is needed as case studies often focus on more than one aspect, measurement site etc. Data in such normal form further facilitates the updating and querying of data, in addition to quality control, i.e., the screening of values for plausibility and errors, that need to be addressed manually.

Second, records are geocoded, and categorial values (re-)coded as necessary. Geocoding, i.e., the conversion of location names to spatial coordinates to map the spatial distribution of analyzed cases, is conducted with the R package *tidygeocoder*, and more specifically through the *Nominatim* geocoding service that is based on OpenStreetMap data [20]. The (re-)coding of categorial data is conducted to address identified issues of data quality, including corrections of mis-inputs, and, e.g., to group rare categories. To facilitate this (re-)coding, categories for recoding with their respective replacement values are collected in text files per predictor variable. The compiled, normalized, and geocoded data is finally controlled for quality. Here, particular care needs to be taken in the context of geocoding, as the geocoding accuracy is dependent on a location's description on the one hand, and on the OpenStreetMap data on the other.

### Presentation layer

The completely processed data is then consolidated to form the data base to be imported by the presentation layer. The dashboard itself is realized as a R Shiny application. Shiny is a package for R that wraps the generation of HTML, CSS and JavaScript code, and that, furthermore, introduces a reactive programming paradigm to track code dependencies for the updating of pieces of information as a reaction to user input [21,22]. Thereby, Shiny allows for a speedy implementation of content-rich, interactive web applications. Due to its embedding into the R statistical language environment, the use of Shiny also facilitates (i) drawing from the rich functionality for handling and visualization of data provided through the R packages repository; and (ii) basing the implementation of the presentation layer on existing workflows in line with the increasing popularity of R, e.g., in the environmental sciences and ecology [23].

**Fig. 2:**
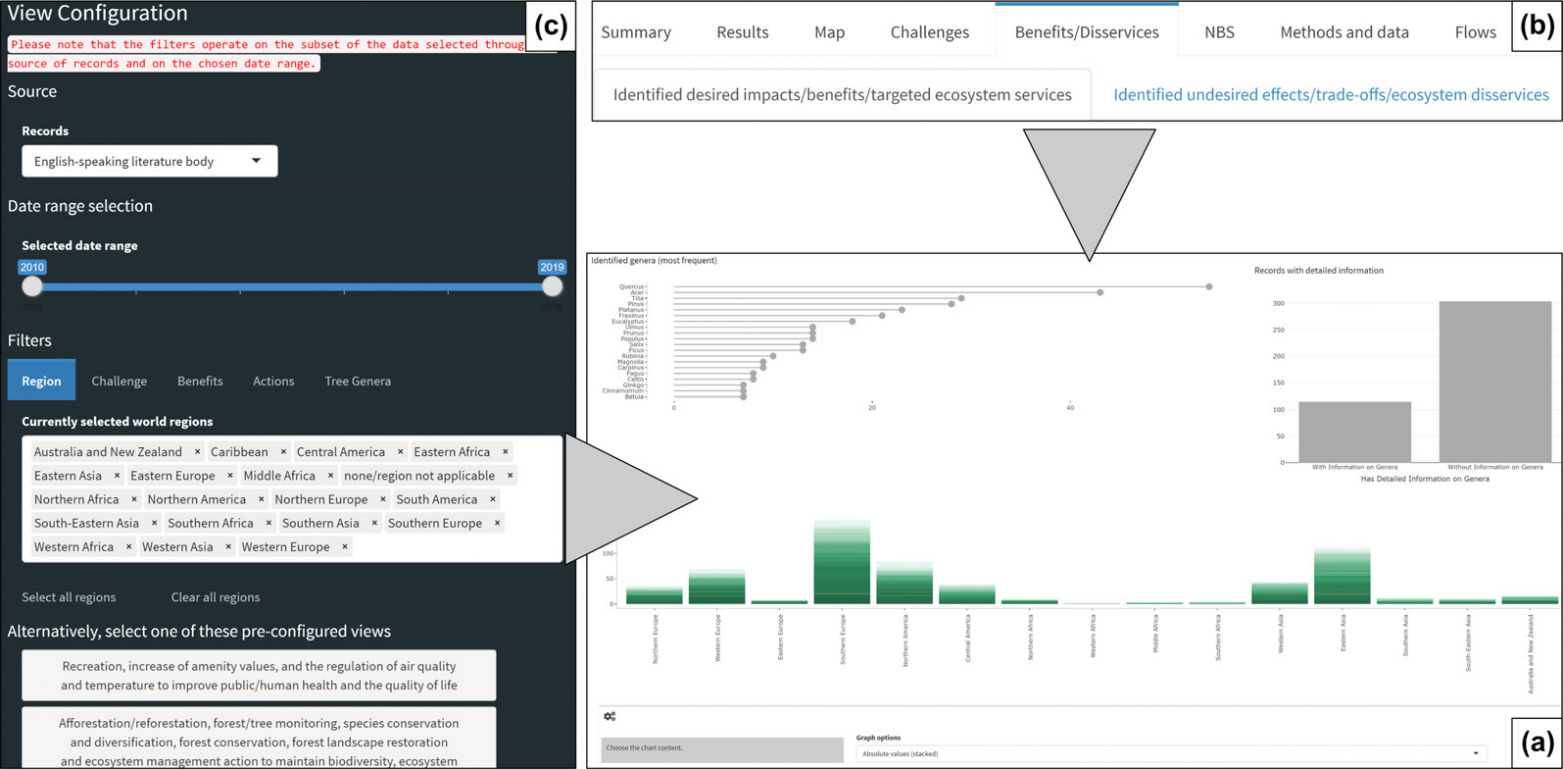
Overview of the dashboard's user interface. The main page (A) embeds the elements used for summarization, narration, and visualization of findings (cf. also Fig. 3 for additional examples). A hierarchical tab interface (B) allows selecting shown data along subjects or themes, with respect to review variables—e.g., challenges, observed benefits, nature-based solutions (NBS), or methods of data (B, top row)—and corresponding (sub-)categories (B, bottom row). Findings can furthermore be filtered and narrowed down by the user through a panel called *view configuration* (C). Available filters include, for example, the year of publication (C, top part), together with various contextual information, e.g., region of study, challenge, observed benefits, nature-based solutions (NBS), or genus of tree (C, middle part). These filtering variables are chosen to reflect on the focus and objectives of the conducted review. Moreover, the dashboard presents the user with a set of pre-defined “*data views*” related to key questions in the context of the CLEARING HOUSE project, including, for example, greening actions in Europe and Eastern Asia, or specific regulation and cultural ecosystem services studied for the improvement of public and human health and well-being (C, bottom part).

The proposed dashboard is realized along a set of functional requirements. A key requirement is to allow a user to perform operations on the available data. Users shall particularly be able to filter and select data with relevance to their interests. This is referred to as defining a *data view* based on a set of pre-defined filter variables, including, e.g., year of publication (Fig. 2).

**Fig. 3:**
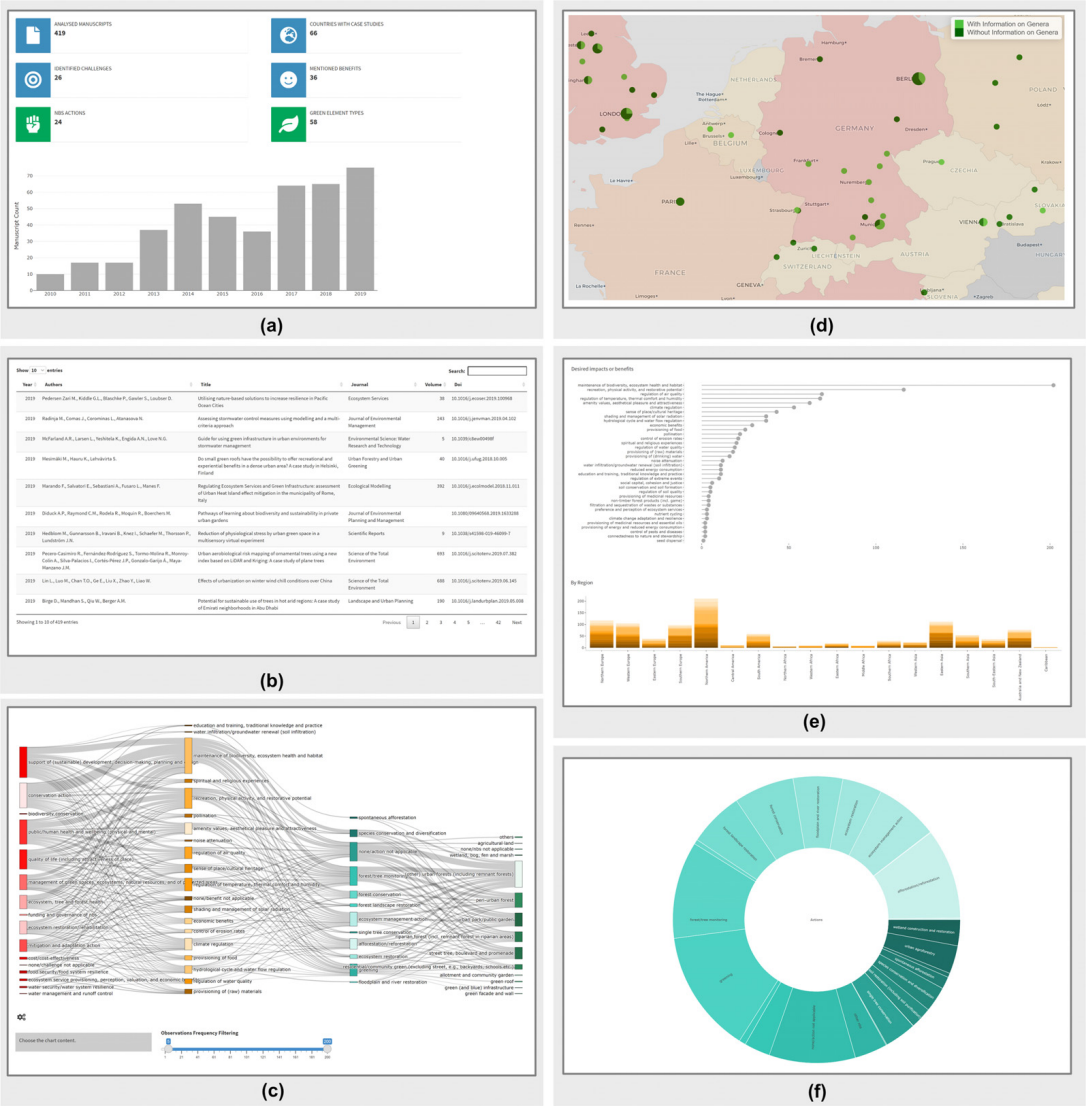
Examples of dashboard elements for the visualization, summarization, and narration of findings. (a) Key indicators for aggregate statistics, e.g., number of relevant records, identified ecosystem services, number of records per year; (b) references, linked to the publication via its DOI; (c) Sankey chart for the summarization of variables, and an at-a-glance overview of relationships between the different categories of reviewed variables; (d) mapping of case studies in the form of a choropleth map combined with local chart signatures; (e) customizable summarizations; (f) use of various chart types across the dashboard, including, e.g., sunburst charts, that allow data drilling.

A further functional requirement is to enable the interactive exploration of data. In line with the dashboards developed in the course of the COVID-19 pandemic, interactive maps are a key component to convey spatial context. Based on the *leaflet* package [24], a choropleth map visualizes the reviewed number of case studies per country. The geocoded case studies are further visualized as local chart signatures—by means of the *leaflet.minicharts* package [25]—that convey contextual, quantitative information. The summarization and narration of findings is supported by rich, customizable data visualizations, including dot plots, column/bar charts, treemaps, heatmaps, sunburst charts, or Sankey diagrams, realized through the R packages ggplot2 and plotly [26,27] (Fig. 3). Data is furthermore summarized in the form of interactive tables, implemented through the *data.table* package [28]. To facilitate customization and interpretation and thus, to accommodate less-experienced users, internal reviews and testing have suggested that a sort of tutorial needs to be integrated. Therefore, a guided tour as along with context-dependent help is implemented through the *cicerone* package [29].

Finally, to promote transparency, the dashboard should enable a more immediate dissemination of findings. This is achieved in two ways: First, data is made accessible and retrievable. This is done, on the one hand, through providing a spreadsheet for download. This spreadsheet is one-hot encoded, i.e., data is provided in a normalized form, so that it can readily be used for querying and statistical analysis. On the other hand, a report document can be obtained that includes maps, charts, (cross-)tables, narrative elements, and references. This document is generated from a parameterized *R Markdown* report [30], so that the document content reflects the filtered data as selected by the user. Second, direct links through the DOI are by directly linking to the analyzed records via their DOI.

### Method validation

As a case, the proposed dashboard integration is exemplified through a SLR conducted in the CLEARING HOUSE project [16] to collate evidence on the impacts and benefits—with respect to key socio-environmental challenges such as biodiversity loss, climate change, disaster risk, and human health and well-being—provided by urban forests and trees within the urban environment as a form of nature-based solutions [17]. In the SLR, a total of 422 records were analyzed with a focus on (i) research context, e.g., societal, economic, or environmental challenges; (ii) geographic context (location and spatial scale); (iii) NBS context, with respect to type of green element or action/intervention studied; (iv) reported benefits and/or trade-offs in regard to specific ecosystem services or ecosystem disservices; and (v) methods and data used. Those aspects were systematically queried and collected in an online spreadsheet in a collaborative manner.

The product is available at https://review.clearinghouseproject.eu. Following the identified key functional requirements, users may apply operations on the data, e.g., data drilling, or by defining data views through the application of filters. These filters include, for instance, study region, contextual challenge, observed benefits, actions, or the tree genus or tree species under investigation. Those filters were chosen with respect to review criteria and in line with the objectives of the CLEARING HOUSE project, thus putting an emphasis on key research aspects such as observed tree benefits in different European or Chinese regions. Moreover, to provoke a deeper interest and establish closer ties with the project's research focus, a set of pre-defined data views is presented to the user in addition to the manual filtering and selection capabilities. These pre-defined views are also formulated along the research questions of CLEARING HOUSE (Fig. 2). The filter criteria presented to the user should consequently be adapted for other reviews. However, based on these criteria, and through the implementation of rich, interactive elements for data visualization, summarization, and narration (Fig. 3), the dashboard supports an exploration of findings, thereby making them accessible.

## Opportunities, challenges, and limitations

The proposed dashboard aims at supporting a rapid dissemination of findings, which is relevant for SLR and other types of literature reviews. In line with synthesizing review findings, dashboards provide means for mapping, visualization, narration, and tabulation of data in a condensed and comprehensive manner. Moreover, although a focus can be put on information most relevant to the accompanying research focus through pre-defined “views”, dashboards nonetheless provide the opportunities to explore generated knowledge through independent and individual analytical lenses. This is contrary to the comparatively constrained synthesis provided in more traditional, (printed) scientific publications. Hence, in so doing, dashboards should be considered as feasible means to increase the impact of literature reviews, with outcomes being made accessible in a more immediate manner, thereby promoting transparency, and additionally contributing to the salience and legitimacy of research that builds on these findings. Transparency might additionally be supported by the methodological need to structure findings for their presentation in a dashboard. On the one hand, this may promote a more-structured data collection already during the actual review. On the other hand, through rigorous and iterative quality control of the findings as part of the data structuring process, flaws in the data may be uncovered.

By conceptualizing the proposed dashboard architecture as separate, loosely coupled layers, an integration into existing, for example R-based data processing workflows is enabled, and iterative updates of the dashboard's data base are facilitated, thereby promoting long-term scientific stewardship [7]. Additionally, transferability and re-use are thus aided. However, particularly a continued maintenance of realized dashboards may pose a significant challenge. In contrast to the manifold repositories available for long-term archival of research data, e.g., *Dryad*, the *UNB Libraries Dataverse Research Data Repository, Zenodo* etc., ensuring a long-term operation of dashboards may be seen as prohibitive with respect to time and/or cost, e.g., considering paid third-party hosting services or regarding staff, especially in the context of the life and funding cycles of academic research projects. A significant barrier may also pose the data science skills needed.

## Conclusion

To support the visualization, narration, and dissemination of findings and data, an integration of dashboards into academic literature synthesis has been proposed. A set of key functional requirements has been identified, including performing operations on data, enabling interactive data exploration, disseminating data directly, and, importantly, providing guidance and assistance to accommodate less-experienced users. The proposed integration is considered useful for a wide range of literature reviews. It may be particularly suitable for SLR or reviews focusing on a large and diverse set of analysis criteria. Contrary to that, the proposed approach is considered less beneficial for theoretical or epistemological research.

## Declaration of Competing Interest

The authors declare that they have no known competing financial interests or personal relationships that could have appeared to influence the work reported in this paper.
